# Lifestyle trajectories in middle-aged adults and their relationship with health indicators

**DOI:** 10.3389/fpubh.2024.1412547

**Published:** 2024-06-05

**Authors:** Alba Roca-Ventura, Javier Solana-Sánchez, Gabriele Cattaneo, Josep M. Tormos-Muñoz, Álvaro Pascual-Leone, David Bartrés-Faz

**Affiliations:** ^1^Institut Guttmann, Institut Universitari de Neurorehabilitació adscrit a la UAB, Badalona, Spain; ^2^Departament de Medicina, Facultat de Medicina i Ciències de la Salut i Institut de Neurociències, Universitat de Barcelona, Barcelona, Spain; ^3^Fundació Institut d’Investigació en Ciències de la Salut Germans Trias i Pujol, Barcelona, Spain; ^4^Departament de Medicina, Universitat Autònoma de Barcelona, Bellaterra, Spain; ^5^Centro de Investigación Traslacional San Alberto Magno, Universidad Católica de Valencia San Vicente Mártir, Valencia, Spain; ^6^Hinda and Arthur Marcus Institute for Aging Research and Deanna and Sidney Wolk Center for Memory Health, Boston, MA, United States; ^7^Department of Neurology, Harvard Medical School, Boston, MA, United States; ^8^Institut d’Investigacions Biomèdiques August Pi i Sunyer (IDIBAPS), Barcelona, Spain

**Keywords:** health, lifestyles, clustering, joint trajectories, k-means, multiple health behaviors, ageing, brain health

## Abstract

**Introduction:**

Understanding the impact of different lifestyle trajectories on health preservation and disease risk is crucial for effective interventions.

**Methods:**

This study analyzed lifestyle engagement over five years in 3,013 healthy adults aged 40-70 from the Barcelona Brain Health Initiative using K-means clustering. Nine modifiable risk factors were considered, including cognitive, physical, and social activity, vital plan, diet, obesity, smoking, alcohol consumption, and sleep. Self-reported diagnoses of new diseases at different time-points after baseline allowed to explore the association between these five profiles and health outcomes.

**Results:**

The data-driven analysis classified subjects into five lifestyle profiles, revealing associations with health behaviors and risk factors. Those exhibiting high scores in health-promoting behaviors and low-risk behaviors, demonstrate a reduced likelihood of developing diseases (*p* < 0.001). In contrast, profiles with risky habits showed distinct risks for psychiatric, neurological, and cardiovascular diseases. Participant’s lifestyle trajectories remained relatively stable over time.

**Discussion:**

Our findings have identified risk for distinct diseases associated to specific lifestyle patterns. These results could help in the personalization of interventions based on data-driven observation of behavioral patterns and policies that promote a healthy lifestyle and can lead to better health outcomes for people in an aging society.

## 1 Introduction

Human life expectancy continues to increase thanks to advances in public health and medicine. The number of adults over age 65 is expected to more than double by the year of 2050 (1). The increase in older aged population is anticipated to result in significant socioeconomic and health care problems as aging predisposes to several chronic diseases that can cause disability and dependency. Advancing age is not only related to increased cognitive decline and dementia (2) but also with depression (3) and cardiovascular events (4). Nonetheless, some individuals age successfully, preserving physical, physiological and cognitive functions as well as sustaining emotional wellbeing (5). Aside from genetic differences, one’s lifestyle can greatly influence overall health and well-being (6), modulating mechanisms of neural plasticity (7), cognitive performance (8) brain resilience (9), and protecting against age-related pathological brain changes and the risk of cardiovascular, neurologic, and psychiatric disorders (10–12). However, individual habits may interact in complex ways and combinations of lifestyle habits—rather than individual factors—may be critical to understand the high inter-individual variability in aging (13). Engagement with lifestyle habits begins in the early stages of life and undergoes variations throughout the vital course (14), influencing the susceptibility to various diseases. Middle-age (40–65 years) is a crucial period, when health problems linked to the cumulative impact of poor health habits frequently start to emerge (15). However, individuals in this age range can potentially benefit from lifestyle interventions, effectively reducing the risk and incidence of chronic conditions, and thereby enhancing their quality of life. Therefore, it is essential to evaluate potential risk factors and their association with diseases well before, in the middle age and late middle age, when the pathological processes are less advanced and optimal preventive effect might be achieved.

To investigate the role of lifestyle choices on healthy aging and risk of new diseases, we leveraged data from the Barcelona Brain Health Initiative (BBHI), an ongoing longitudinal prospective cohort study examining the lifestyle factors, biological determinants, and their interactions, related to mental and brain health maintenance in aging (16). BBHI focuses on the following health behaviors given their relevance for brain health (9, 12, 17): cognitive activity, physical exercise, sleep, socialization, nutrition, vital plan, and general health.

## 2 Materials and methods

### 2.1 Participants and measures

The BBHI started with the aim to understand and characterize the factors related to brain health maintenance in middle-aged adults. The BBHI involves 5,468 participants aged 40–65 years (67.2% women) free from any psychiatric or neurological major diagnoses made by their reference physicians at the time of recruitment. Participants are community-dwelling individuals, with more prevalence of women, highly educated, and with better lifestyles compared with the general population (16, 18).

**Figure 1:**
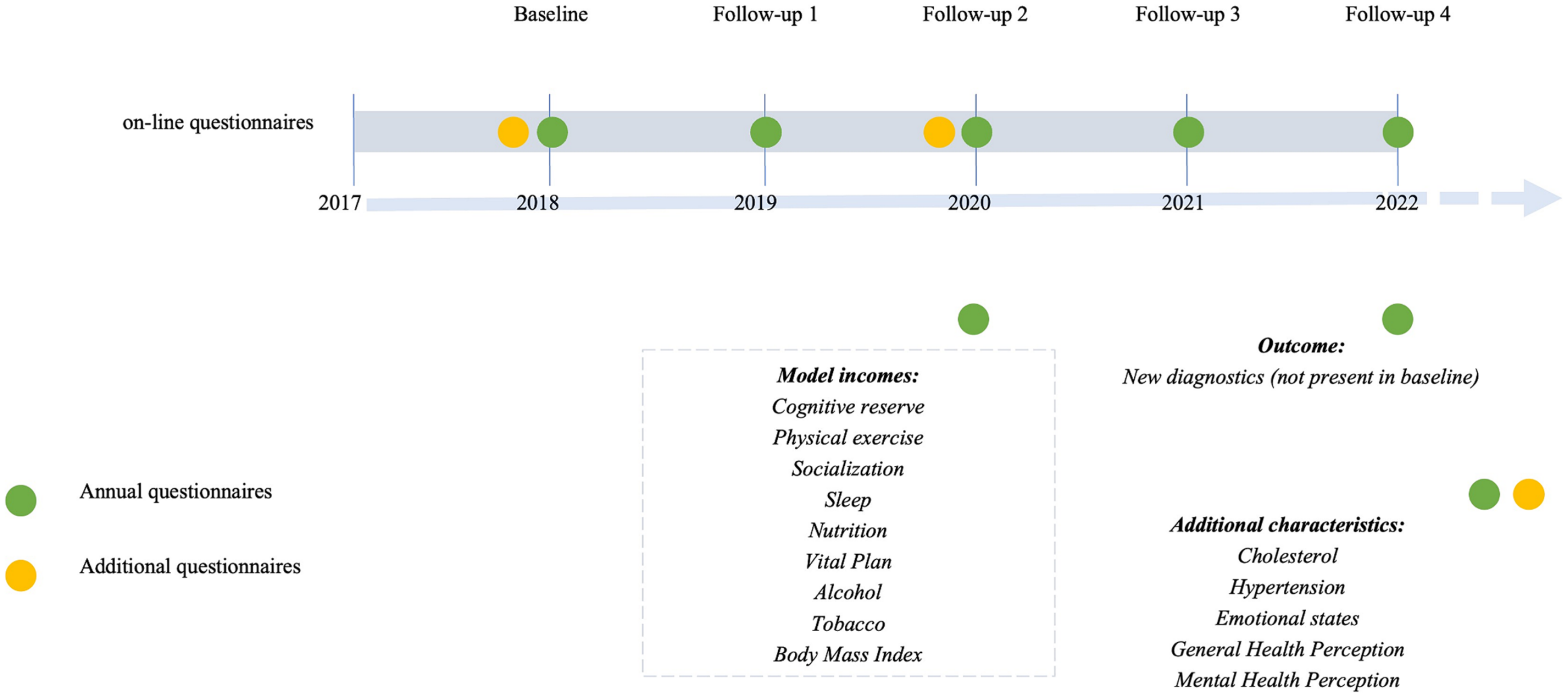
Barcelona brain health initiative timeline.

The data used in the present study was obtained from on-line annual follow-ups conducted between 2018 and 2022. Only participants who completed three or more yearly assessments, and had no major diagnoses at the time of recruitment (List of considered diagnoses in Supplementary Table S2) were included in this analysis (*n* = 3,013; 66.4% women). At each follow up, participants were asked to complete several on-line validated questionnaires (Table 1) (16, 18) to systematically collect information about habits and lifestyle, psychological and emotional well-being. Self-administered questionnaires measured nine components of modifiable healthy lifestyles were included in the model (Figure 1). Six of them correspond to BBHI pillars: Cognitive activity, physical activity, sleep, nutrition, vital plan, and socialization; and other three risk factors including alcohol consumption, tobacco use and Body Mass Index (BMI). In addition, participants were required to report any new diagnoses, provide an assessment of their general and mental health perception, and specify health indicators such as cholesterol and hypertension at each follow-up. Moreover, additional questionnaires related to emotional states were completed at two different time points (detailed information of questionnaires is available in Supplementary material).

**Table 1:** Descriptive analysis across five lifestyle trajectory group in the longitudinal lifestyle data.

			A. Healthy	B. Low cognitive reserve	C. Obesogenic	D. Heavy smokers	E. Alcohol-sleep	Differences between groups
		N	1,249	908	432	300	124	
		%	41.4%	30.1%	14.3%	10%	4.1%	
Age			57.5 (7.18)	56.5 (7.23)	57.8 (6.87)	**60.1 (6.38)**	55.7 (6.28)	Tukey Contrasts *p* < 0.001*
Gender	Men	N	337	332	153	117	72	χ^2^: 66 (4) *p* < 0.001 Cramer: 0.15 (small)
		%	27	36.6	35.4	39	58.1	
	Women	n	912	576	279	183	52	
		%	73	63.4	64.6	61	41.9	
Education	Primary	n	16	47	17	19	2	χ^2^:124 (8) *p* < 0.001 Cramer: 0.14 (small)
		%	1.3	5.2	3.9	6.3	1.6	
	Secondary	n	194	263	105	98	37	
		%	15.5	29	24.3	32.7	29.8	
	Superiors	n	1,039	598	310	183	85	
		%	83.2	65.9	71.8	61	68.5	
Marital status	Married	n	776	572	266	158	69	χ^2^: 29.5 (12) *p*: 0.003 Cramer: 0.06 (very small)
		%	62.1	63	61.6	52.7	55.6	
	Separated or divorced	n	213	143	69	63	19	
		%	17.1	15.7	16	21	15.3	
	Widowed	n	30	13	14	15	1	
		%	2.4	1.4	3.2	5	0.8	
	Single	n	230	180	83	64	35	
		%	18.4	19.8	19.2	21.3	28.2	

*The bold values wanted to reflect the value with a significant difference from the others.

### 2.2 Statistical analyses

Analyses were conducted in R version 2022.07.1. To estimate the joint trajectories of lifestyles, K-means for longitudinal data with the KmL3D package were calculated (19, 20). KmL3D is an updated version of the popularly used K-means clustering algorithm adapted to enable longitudinal clustering analysis with several variables repeatedly measured over time (called joint trajectories). This method is shown to be appropriate for studying the joint evolution/variation and complex interactions between several variables over time. Assuming that the trajectories are interconnected, KmL3D clusters the data based on the combined distances between the variable’s trajectories into K user-defined disjointed clusters so that the participants within the same group exhibit maximum pairwise similarity scores and high dissimilarity scores with the participants belonging to other cluster groups (19). To evaluate the optimal number of cluster trajectories, we tested several models (see Supplementary Table S3). Each model was repeatedly fitted with the number of clusters increasing stepwise from 2 to 5 using maximum likelihood criterion, computed using the KmL3D algorithm. We selected the best solution based on different model-fitting criteria, due to its parsimony, BIC, and because cluster number choice revealed the most meaningful clustering for health behaviors. By limiting the study to those who completed three or more annual assessments we kept the proportion of missing data lower than 40% and reduced the potential for bias (21) (see Supplementary Table S4). Missing items for the included samples were imputed by the *CopyMean* imputation method (19) to reduce the bias and variance in the results. Successful imputation was confirmed by descriptive analyses and comparison of means.

Baseline sociodemographic characteristics were compared between clusters. Additionally, the means and standard deviations of the trajectories were calculated for each questionnaire included in the model, as well as for the additional questionnaires. Kruskal-Wallis and *post-hoc* analyses were used to compare the results between clusters.

To determine whether there was a difference in the distribution of newly developed diagnoses among the data-driven clusters, Kaplan–Meier cumulative-incidence curves were constructed using the Cox model separately for each cluster, and clusters statistically compared with chi-squared test. We categorized diagnoses into four groups: all diagnoses, psychiatric (anxiety and depression), neurological (Parkinson’s disease, Alzheimer’s disease, and Mild Cognitive Impairment) and cardiovascular (Cerebral infarction, Heart attack, Heart problems). The list of diseases used in the analysis can be found in Supplementary Table S2.

## 3 Results

### 3.1 Joint developmental trajectories lifestyles

Participants were middle aged (57.4 ± 7.1 years), well educated (71% high education), and 68.3% were women.

**Figure 2:**
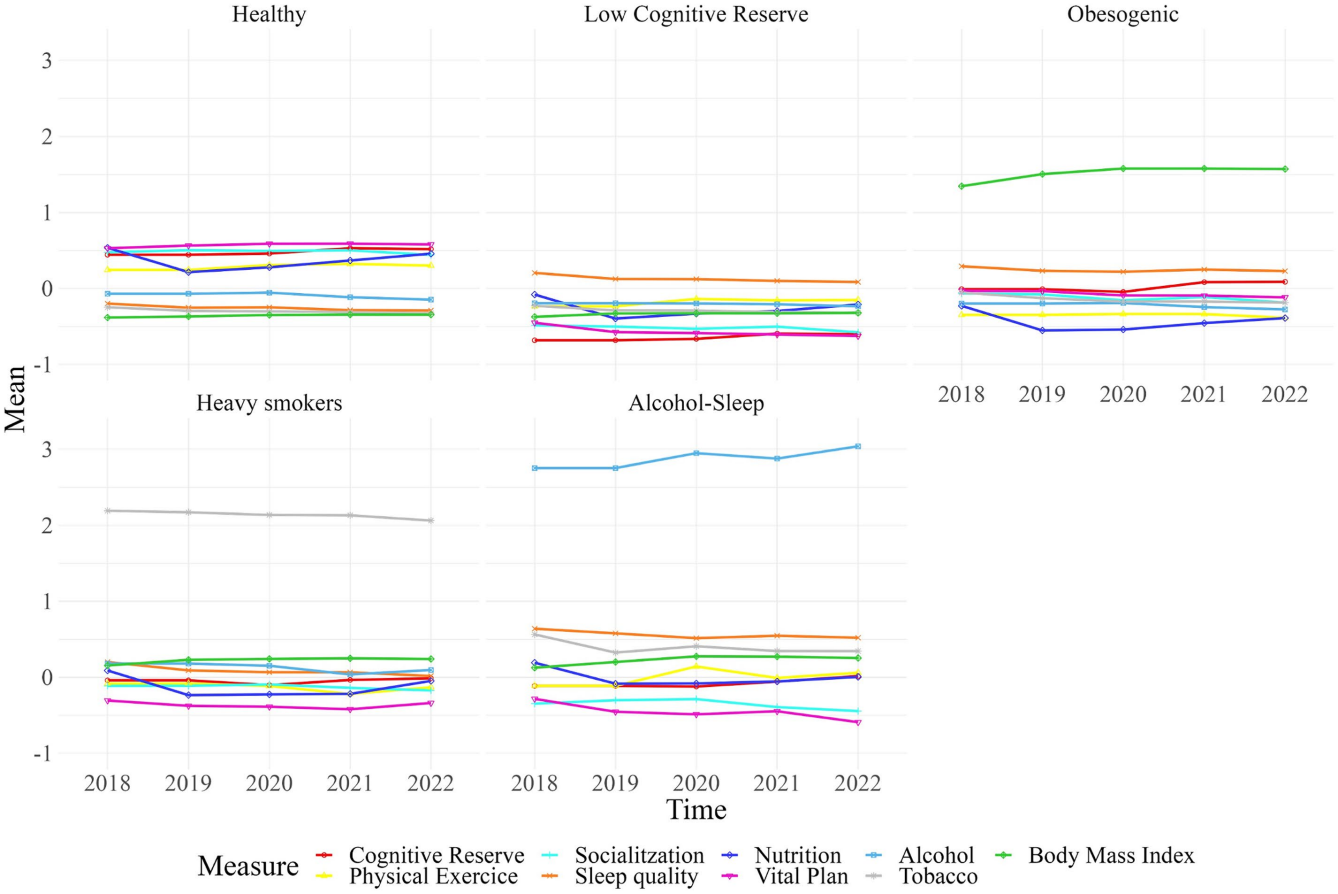
The five joint trajectories of healthy habits. Each line represents one of the lifestyle input variables used to generate clusters.

The clustering methodology identified five joint trajectories of lifestyles which we labeled based on the main characteristics as follows: (A) Healthy, (B) Low Cognitive Reserve, (C) Obesogenic, (D) Heavy smokers, and (E) Alcohol-Sleep. Figure 2 shows the pillar trajectories for each cluster.

The “A. Healthy” trajectory (*n* = 1,249, 41.4%), includes adults characterized by exhibiting high scores in cognitive activity, nutrition habits, physical exercise, socialization and vital plan, low scores in tobacco use, good sleep quality and normal body mass index, otherwise alcohol consumption was considered low risk. The “B. Low Cognitive Reserve” trajectory (*n* = 908, 30%) includes adults with low ratings in the Cognitive Reserve Questionnaire and low scores in socialization and Vital Plan scales and moderate to low scores in sleep quality. This group has healthier scores in alcohol, smoking, and Body Mass Index (BMI) compared to other groups. The “C. Obesogenic” trajectory (*n* = 432, 14.3%) is characterized by high BMI and poor nutrition and exercise habits, with a mean BMI of 31.84 considered obese by World Health Organization (22). The “D. Heavy Smokers” trajectory (*n* = 300, 10%) is mainly distinguished by its high smoking habits (two deviations from the mean), though other factors, such as low Vital Plan scores, cholesterol, hypertension, and moderate to unfavorable outcomes on the other scales, are also representative. The “E. Alcohol-Sleep” trajectory (*n* = 124, 4.1%) is distinguished by harmful alcohol consumption as well as poor sleep quality. This group also has low scores in well-being, quality of life and meaning in life, general and mental health perception, and socialization.

Baseline sociodemographic characteristics were compared between the five clusters in Table 1.

Table 2 employs a gradient color scheme (red-yellow-green) to visually represent the means of the trajectory for each scale utilized in the model within each cluster. In this gradient, red indicates poorer results, while green signifies better outcomes for each health behavior. Supplementary Figures S1–S9 provides detailed results from Kruskal-Wallis and post-hoc analyses for each questionnaire, highlighting significant differences observed across clusters.

**Table 2:** Means and standard deviation of the trajectory of each questionnaire included in the model by cluster.

		A. Healthy	B. Low cognitive reserve	C. Obesogenic	D. Heavy smokers	E. Alcohol-sleep
**Modifiable factors added to the model**						
Cognitive reserve	CRQ	15.51 (2.53)	11.45 (3.11)	14.01 (3.09)	13.61 (3.33)	13.13 (3.78)
Physical exercise	IPAQ Mets	3,319.59 (2,100.08)	2,444.91 (1,660.41)	2069.73 (1,695.47)	2,550.61 (1823.55)	2,841.37 (2092.48)
Socialization	Lubben	40.3 (6.25)	31.2 (7.65)	35.14 (8.02)	34.58 (7.54)	33.66 (7.77)
Sleep	Jenkins	7.31 (2.53)	8.67 (3.18)	9 (3.65)	8.33 (3.07)	9.97 (3.7)
Nutrition	Medas	9.18 (1.45)	8.02 (1.56)	7.71 (1.42)	8.23 (1.66)	8.34 (1.74)
Vital plan	RYFF	57.69 (5.59)	47.62 (6.35)	52.39 (6.95)	49.26 (7.58)	49.83 (7.2)
Alcohol	Audit	2.22 (1.44)	1.95 (1.44)	1.88 (1.51)	2.71 (1.59)	8.76 (2.83)
Tobacco	PxY	2.88 (5.06)	2.68 (4.67)	4.47 (7.04)	29.84 (11.54)	11.72 (11.64)
Body mass index	BMI	23.65 (2.75)	23.62 (2.66)	31.84 (3.74)	26.16 (3.96)	26.19 (3.89)

CRQ, Cognitive Reserve Questionnaire; IPAQ, International Physical Activity Questionnaire; PxY, Pack per Year (detailed information of questionnaires is available in Supplementary material). Color gradient description. CRQ: no cut-off, more score in green, less score in red. Physical exercise: based on the category of the IPAQ, from green that represents category high, yellow/orange moderate to the low category in red. Lubben: no cut-off, gradient from the highest score indicating more social engagement in green and less engagement in red. Jenkins: no cut-off, gradient from the lower score indicating less sleep problems in green and greater number of sleep problems in red. Medas: no cut-off. Gradient from green meaning higher score to red meaning worst score. Ryff: no cut-off. Gradient from green represent more psychological wellbeing to red for lower scores. Audit: in red more than 8 is considered harmful consumption. Gradient of green for less consumptions. PxY: green a smaller number of packages of tobacco per year. For instance, a person who smokes one pack of cigarettes every day for 30 years is a 30-pack year smoker, 25 cigarettes per day is considered heavy smoker. BMI: in red more than 30, obesity; 25–29.9 overweight in yellow and 18.5–24.9 normal weigh in green.

Additional questionnaires were also compared between clusters using the means and standard deviation of each variable, as well as the percentage of new cases for cholesterol and hypertension. The results can be observed in Table 3, utilizing the same gradient color scheme.

**Table 3:** Means and standard deviation of additional questionnaires score and percentage of incidence of cholesterol and hypertension by cluster.

		A. Healthy	B. Low cognitive reserve	C. Obesogenic	D. Heavy smokers	E. Alcohol-sleep
General health	PROMIS	36.68 (4.28)	33.31 (4.94)	32.75 (5.31)	33.2 (5.18)	32.3 (4.98)
Cognitive complains	PROMIS CA and CC	44.62 (4.35)	40.48 (6.6)	42.09 (6.08)	41.48 (6.5)	38.7 (6.48)
Anxiety and depression	DASS depression	2.71 (3.86)	6.23 (6.54)	5.38 (5.95)	5.52 (6.38)	7.77 (8.33)
	DASS anxiety	2.44 (3.26)	4.15 (5.05)	3.98 (4.47)	4.2 (5.29)	5.9 (5.92)
	DASS stress	7 (5.79)	10.23 (7.22)	9.55 (6.88)	10.01 (7.24)	12.48 (8.25)
	DASS total	12.15 (10.93)	20.61 (16.15)	18.92(14.82)	19.73 (16.31)	26.15 (19.88)
Cardiovascular risk factors	Hypertension	12.9%	17.5%	38%	31%	21.8%
	Cholesterol	31.8%	33.7%	35.2%	47.7%	43.5%

PROMIS, Patient-Reported Outcomes Measurement Information System; DASS, Depression, Anxiety and Stress Scale.

### 3.2 Relationship between healthy habits and the probability to develop cardiovascular, neurological, and psychiatric diseases

**Figure 3:**
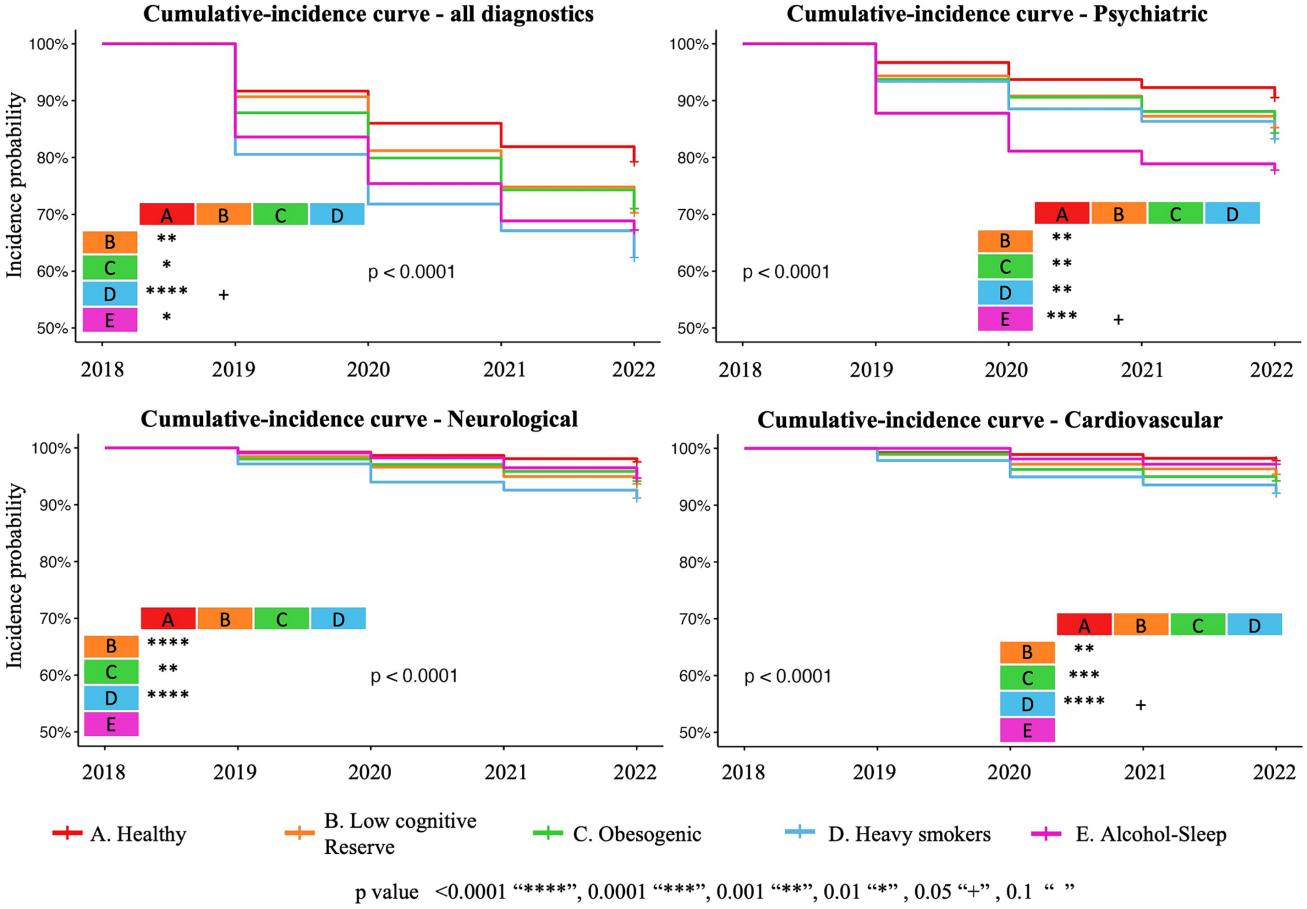
Graphical representation of the cumulative-incidence curves for all new diseases, psychiatric, neurological, and cardiovascular diseases based on a Cox proportional hazards model.

Diagnoses were categorized into four groups: all diagnoses (28.22%), psychiatric (11.64%), neurological (5.7%) and cardiovascular (3.5%). Cumulative-incidence curves analysis, depicting the incidence of new diagnoses, is presented in Figure 3. Compared to group A, participants in the other four groups exhibited higher cumulative-incidence rates of diseases (*p* < 0.0001) across all diagnostics comparison. Additionally, group D also showed a significantly worse cumulative-incidence curve compared to group B (*p* = 0.05). Different lifestyle clusters were differentially associated with various pathologies, as illustrated in Figure 3.

**Figure 4:**
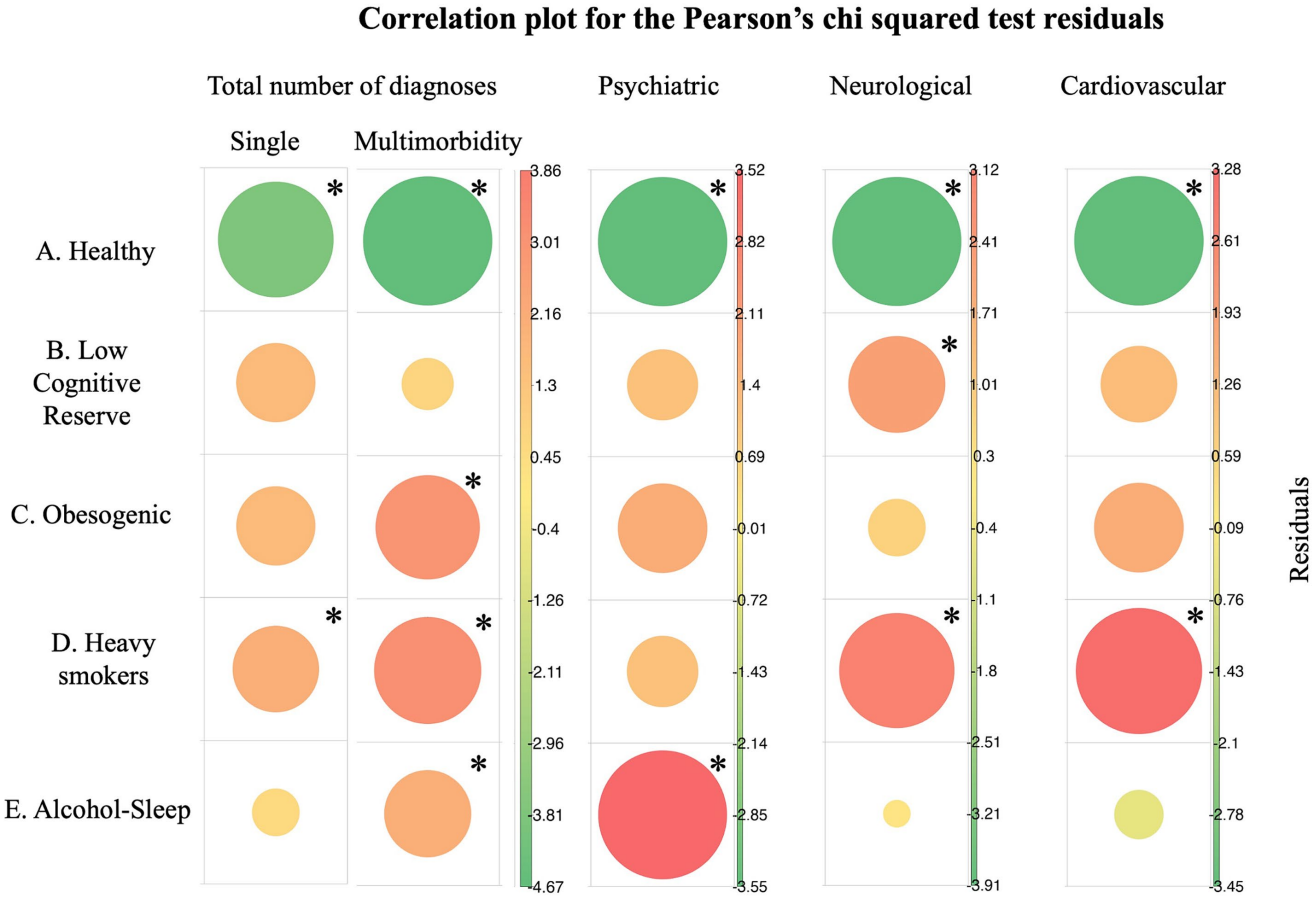
Pearson’ residuals visualization for total diagnostics, psychiatric, neurological, and cardiovascular diseases. In green negative residuals, suggesting negative association which implies protection from diseases and in red positive residuals meaning increased risk associations. The size of the circle depicts the cell contribution to the total Chi-square (bigger circles mean stronger association). Single morbidity refers to individuals with one disease, while multimorbidity refers to individuals with two or more diseases. *In asterisks the major and most significant contribution of the cell on the significant chi-square test statistic.

The results from chi-square analysis are shown in Figure 4 and demonstrate the association between each identified lifestyle trajectories cluster and the number of new diseases for: total number of diagnoses both single and multimorbidity cases (χ^2^ = 99.276, df = 8, *p* < 0.001), psychiatric diseases (χ^2^ = 25.281, df = 4, *p* < 0.001), neurological diseases (χ^2^ = 14.244, df = 4, *p* < 0.01), and cardiovascular diseases (χ^2^ = 25.377, df = 4, *p* < 0.001).

## 4 Discussion

In this study, we examined joint trajectories of nine lifestyle factors during 5 years in a cohort of healthy middle-aged adults. We found that specific modifiable lifestyle factors tend to cluster together, resulting in different profiles characterized by distinctive attributes. These profiles provide valuable insights into the development of various diseases. This knowledge may enable targeted interventions and empower individuals to take proactive steps toward optimizing their brain health and aging.

Our study has some limitations. First, the data in this study was mostly self-reported. Second, aggregation of diseases into one sum score is a simplification and may obscure the relative relevance or weight of the different diseases. Finally, as previously described in Cattaneo et al. (16) participants from the BBHI were volunteers with slightly higher representation from affluent groups, highly educated and with more women; living with a partner and children is higher than in the general population (48.8% vs. 33.6%; data from the Statistical Institute of Catalonia).^1^ In line with previous results (18) participants who completed the follow-ups were slightly older. Therefore, participants may not be completely representative of the general population. Similarly, this study is not focused on the early and late life stages critical for understanding aging.

The data-driven analysis identified five groups (healthy, low cognitive reserve, obesogenic, heavy smokers, and alcohol-sleep-). Moreover, the findings showed that the lifestyle joint trajectories within the identified clusters remained stable throughout time, which is consistent with past studies that found clusters to be stable at different phases of life in the absence of interventions aimed at habit changes (23).

Not surprisingly, the group with the highest levels of all health-promoting habits exhibited reduced risk of chronic diseases, and overall optimal perceived general and mental health. Conversely, when the adherence to health-promoting behaviors was relatively lower, more negative emotional symptoms and risk of chronic diseases tend to appear, these results align with previous literature (23, 24). Two systematic reviews have already studied the clustering or co-occurrence of modifiable risk factors. Noble et al. (25) investigated clustering studies involving four risk behaviors: smoking, poor nutrition, excessive alcohol consumption and physical inactivity. Similar to our research, a healthy cluster, characterized by the absence of any target risk factor was identified in numerous studies. Moreover, the co-occurrence of alcohol and smoking cluster, the poor diet and low activity, as well as clusters where all the risk factors studied tended to co-occur, were prevalent. Our data-driven analyses further distinguished various aggregations of lifestyle behaviors, providing a nuanced perspective beyond the all-risk profile identified in the literature review, with the more potential to target the intervention of lifestyle habits. Concerning the co-occurrence of alcohol and smoking, in our study two different clusters were identified and particularly the “Alcohol-Sleep” cluster (Cluster E) exhibited a tendency to consume both substances, although the two clusters showed different characteristics and different prediction risk to diseases. In Cluster E the adherence to unhealth behaviors leads to a moderate risk of all diagnostics incidence and high risk to psychiatric diseases. This group also exhibited poor general and mental health perception and high levels of anxiety and depression. On the other hand, “Heavy smokers” group (Cluster D) were highly susceptible to various health conditions, particularly cardiovascular and neurological diseases, underscoring the detrimental effects of smoking on health (26). The co-occurrence of poor diet and low activity was also present in the “Obesogenic” group (Cluster C). Moreover, this group showed an increased susceptibility to multimorbidity and cardiovascular diseases, alongside elevated BMI and cholesterol. These findings underscore the detrimental impact of obesity on health, a trend consistent with observations in other studies. Notably, maintaining a normal weight has been particularly important for those who achieved higher ages without chronic diseases (24). Studies focusing on disease clustering reveal a distinct cardiometabolic profile (27), wherein various risk factors tend to aggregate. Importantly, these profiles exhibit stability from childhood into adulthood. The cluster found in our study offers valuable insights for characterizing high-risk individuals, illuminating potential intervention strategies aimed at mitigating the progression of cardiovascular diseases at an early stage (28).

Meader et al. (29) conducted an analyses of studies examining the co-occurrence or clustering of two or more risk behaviors, not limited to diet, physical activity, alcohol or smoking. This systematic review indicated that, among general adult populations, alcohol misuse and smoking constituted the most commonly identified risk behavior cluster. Among young adults, consistent evidence of clustering was observed between sexual risk behavior and substance misuse. However, it was noted that most studies used a generic measure comparing the engagement in any risk behavior with the absence of risk behavior. This board approach, encompassing any two or three risk behaviors, lacked the granularity to identify specific patterns, as demonstrated by our in-depth analyses. Notably, our analyses revealed patterns of aggregation, contributing to a more comprehensive understanding of the relationship between lifestyle behaviors and its implication for disease development. Furthermore, none of the studies reviewed, including recent ones, explored the clustering of cognitive activity, socialization, sleep and vital plan—lifestyle factors that appear to be highly significant (9) and demonstrate a tendency to co-occur with other important variables in our study. For instance, Cluster B, characterized by subjects with low cognitive activity as well as poor socialization and vital plan, exhibits associations with neurological diseases. A targeted intervention aimed at enhancing cognitive activity, socialization and vital planning within this cluster could potentially mitigate the risk of developing such neurological diseases.

Recently, Jia et al. (30) introduced a categorization framework based on the number of healthy lifestyle factors adhered to, delineating unfavorable group for those following 0 or 1 factor, an average group for 2–3 factors, and a favorable for 4–6 factors. This classification has demonstrated associations with memory decline, even in the presence of APOE ε4 allele, when comparing the unfavorable group against both the favorable and average groups. In our study, the Cluster A, equivalent to Jia et al. favorable group, similarly detects better results in health outcomes compared to other clusters that follow more than one unhealthy behavior and they could be equivalent to the unfavorable or the average group of Jia and collaborator’s classification. Nonetheless, our analysis has also identified profiles characterized by the coexistence of healthy and unhealthy behaviors, akin to Jia et al. (30) average group, which confer a risk for disease development. These findings provide crucial insights, highlighting that individuals often adopt a mix of behaviors that transcend binary categorizations of healthiness. While such profiles exhibit healthier practices alongside unhealthy habits, they remain susceptible to disease in midlife, potentially culminating in increased risks of dependency, disability, and mortality with advancing age.

By leveraging this knowledge, public health professionals, policymakers, and healthcare providers could tailor interventions and design targeted strategies to promote positive lifestyle changes, leading to more effective and efficient allocation of resources and interventions, resulting in improved health outcomes and enhanced quality of life for people. Multimodal interventions simultaneously targeting multiple risk factors and disease mechanisms are more likely to be effective (29). Recent multidomain trials, while exhibiting mixed outcomes in unselected population, have shown improved results when preventive interventions are specifically directed toward at-risk individuals (31). These results, along with our findings suggest that not only multimodal interventions are crucial for changing lifestyles habits and improving health outcomes, but it is also important to recognize that interventions need to be personalized and target profiles as specific lifestyle patterns have been associated to risk for distinct diseases. Indeed, implementing uniform approaches is not effective in promoting sustained behavior change considering the persistent nature of these habits, interventions should be designed to target specific lifestyle profiles and address the unique challenges and barriers associated with each profile (23, 25, 29, 32). By tailoring interventions to individuals’ needs and providing ongoing support, it becomes more feasible to introduce and maintain positive lifestyle changes over time (33). For instance, personalized interventions could focus on individuals within specific profiles with potential risks associated not only with their current lifestyles but also with the diseases they could develop, while offering tangible steps to adopt healthier habits.

In conclusion, the combination of valuable insights from this study and the utilization of joint trajectories cluster analysis holds immense potential in driving forward the field of brain health maintenance and promotion. With this knowledge, we can design evidence-based interventions that not only raise awareness and encourage the adoption of brain-healthy behaviors but also consider the intricate relationships between different variables, thereby improving the overall effectiveness of brain health initiatives in the population.

## Data availability statement

The datasets for this article are not publicly available due to concerns regarding participant anonymity. Requests to access the datasets should be directed to JS-S (jsolana@guttmann.com) or DB-F (dbartres@ub.edu).

## Ethics statement

The studies involving humans were approved by Comité d’Ètica I Investigació Clínica de la Unió Catalana hospitals, CEIC18/07. The studies were conducted in accordance with the local legislation and institutional requirements. The participants provided their written informed consent to participate in this study.

## Author contributions

AR-V: Data curation, Formal analysis, Investigation, Methodology, Validation, Visualization, Writing – original draft, Writing – review & editing. JS-S: Conceptualization, Data curation, Funding acquisition, Methodology, Project administration, Software, Supervision, Validation, Writing – original draft, Writing – review & editing. GC: Formal analysis, Funding acquisition, Methodology, Project administration, Supervision, Validation, Writing – original draft, Writing – review & editing. JT-M: Conceptualization, Funding acquisition, Writing – review & editing. ÁP-L: Conceptualization, Funding acquisition, Validation, Writing – original draft, Writing – review & editing. DB-F: Conceptualization, Funding acquisition, Resources, Supervision, Validation, Writing – original draft, Writing – review & editing.

## Footnotes

^1^ www.idescat.cat
