# Supplementary material for: Lifestyle trajectories in middle-aged adults and their relationship with health indicators
Source: Front Public Health. 2024 Jun 5;12:1412547. doi: 10.3389/fpubh.2024.1412547 (PMC11188459; doi:10.3389/fpubh.2024.1412547)
Supplement: Supplementary file 1 [file Data_Sheet_1.pdf]

Supplementary Material

1 Supplementary Data

Table S1. list of questionnaires administered.

QUESTIONNAIRE		B	F1	F2	F3	F4
	**YEARLY QUESTIONNAIRES**					
**Sleep***	Jenkins Sleep Evaluation Questionnaire (JSEQ)(1)	●	●	●	●	●
**Nutrition***	Mediterranean Diet Adherence screener (MeDas)(2)	●	●	●	●	●
**Vital plan***	Personal growth and Purpose in life subscales of Ryff's scale of Psychological wellbeing (3)	●	●	●	●	●
**Smoking***	Pack-year index	●	●	●	●	●
**Socialization***	Lubben Social Network Scale (LSNS)(4)	●	●	●	●	●
**Cognitive reserve***	Cognitive Reserve Questionnaire (CRQ, (5)		●	●	●	●
**Alcohol consumption***	AUDIT (6)		●	●	●	●
**Physical activity***	International Physical Activity Questionnaire (IPAQ)(7)		●	●	●	●
**Self-perceived general health**	PROMIS (8)	●	●	●	●	●
**Self-perceived mental health**	PHQ-4 (9)	●	●	●	●	●
**Cognitive complaints**	PROMIS® Cognitive Abilities and Cognitive Concerns (10)	●	●	●	●	●
**General health**	Diseases	●	●	●	●	●

	Body Mass Index*	Height(m)	●	●	●	●	●
		Weight (kg)	●	●	●	●	●
**Additional questionnaires**							
**Emotional health**	Depression Anxiety and Stress Scale (DASS; (11)		●		●		

*Questionnaires used as input variables in the clustering model

B: Baseline; F1: Follow-up 1; F2: Follow-up 2; F3: Follow-up 3; F4: Follow-up 4

1.1 Input Variables to de model

1.1.1 Cognitive Reserve

Cognitive reserve was assessed using the Cognitive Reserve Questionnaire (CRQ; (5) which has been validated in the Spanish population both in healthy subjects and those with early stages of Alzheimer's disease. It is composed of eight items that address engagement in cognitively stimulating activities, such as participants and partents' academic and courses training, professional occupation, music training and languages proficiency, and it takes a few minutes to complete. To obtain the total score the results of each item are added up to a maximum score of 25 points. Higher scores imply greater estimations of cognitive reserve.

1.1.2 Physical Exercise

Physical activity was assessed with the self-reported International Physical Activity Questionnaire (IPAQ;(7). IPAQ identifies the frequency and duration of moderate and vigorous leisure, transportation, and occupational physical activity, walking physical activity, and inactivity during the past week. For each question, participants are given examples of moderate, vigorous, and walking activities, and physiological cues for breathing and the heart rate to help them recall activities at an appropriate intensity level. Activities were afterward transformed to total score (Metabolic Equivalent of Task; MET) and also classified into light, moderate, and vigorous intensity, according to the guidelines for data processing and analyses of the IPAQ (12).

1.1.3 Socialization

Social support networks were assessed by the revised Lubben Social Network Scale (LSNS; (4). It is a brief instrument designed to capture social isolation in older adults by measuring perceived social support from family, friends, and neighbors in terms of the size, closeness and frequency of contacts of a respondent's social network (13). The total score is the sum of all items. For the LSNS, the score ranges between 0 and 60, with a higher score indicating more social engagement.

1.1.4 Nutrition

To determine the degree of adherence to the Mediterranean Diet, the MEDAS-14 questionnaire was used (2). It is composed of 14 questions, 12 questions regarding the frequency of consumption and 2 regarding intake habits characteristic of the Spanish Mediterranean Diet. Each question is scored 0 or 1. From the sum of the values obtained for the 14 items, score ranged from 0 to 14, the degree of adherence to the Mediterranean Diet is determined and used in the analysis.

1.1.5 Vital Plan

To evaluate Vital plan, the Personal Growth and Purpose in life subscales of Ryff's scale of Psychological well-being were administered. The Ryff is a straightforward and relatively short survey that assesses the psychological component of well-being. Respondents rate statements on a scale of 1 to 6, with 1 indicating strong disagreement and 6 indicating strong agreement (3). Both subscales have 7 items each making a total score from 14 to 84.

1.1.6 Sleep

Jenkins Sleep Scale (JSS,(1) is an efficient instrument for the evaluation of multiple sleep complaints. JSS is a simple, self-reported, brief scale. The questionnaire consists of four items that evaluate sleep difficulties experienced in the past four weeks. It includes inquiries about difficulties in initiating sleep, difficulties in maintaining sleep, frequent awakenings during the night, and subjective experiences of fatigue and sleepiness despite having a typical night's sleep (14) The respondents answer the questions using a 6-point Likert-type scale from 0 (not at all) to 5 (22 to 31 days). The total scores range from 0 to 20, and higher scores indicate a greater number of sleep problems (1).

1.1.7 Obesity

We used Body Mass Index (BMI) as a proxy to measure an individual's obesity. Although other variables such waist circumference or percent body fat could be used to measure obesity precisely, BMI is the most commonly used physical marker to assess obesity in similar articles. Weight and height were collected at every wave and used for calculating the BMI, by dividing the weight (kg) by the height squared (m2).

1.1.8 Alcohol Consumption

The World Health Organization's Alcohol Use Disorders Identification Test (AUDIT) is the most widely tested instrument for screening alcohol consumption. In addition to questions about alcohol use, the AUDIT also asks about common alcohol-related problems that patients may experience as well as common symptoms of alcohol dependence, and these responses are incorporated into the patient's total score. Responses to each question are scored from 0 to 4 and the total score range from 0 to 40 (6), 8 or more in the total score indicates a strong probability of damage due to alcohol consumption.

1.1.9 Tobacco

The degree of lifetime exposure to tobacco smoking was assessed as pack-years (15–18), calculated by multiplying the years of smoking with the self-reported number of smoked cigarettes per day. This smoking pack year calculation helps to produce a numerical value of lifetime tobacco exposure.

1.2 Outcomes

1.2.1 Diagnoses

At each online follow-up, participants were asked to report new medical conditions that had been diagnosed by a physician during the previous year (see supplementary material for the whole list). Participants with self-reported diseases prior to the study were excluded from the analysis and only new diagnosis were considered.

1.2.2 Additional Information

Additional economic and sociodemographic information was gathered during each follow-up, and supplementary questionnaires including the International Personality Item Pool – IPIP (19) and the Depression Anxiety and Stress Scale (DASS; (11) were administered on-line at the second follow-up and at the baseline and second follow-up respectively. This information enabled the examination of additional correlated factors, encompassing personality traits, emotional well-being, and participants' socio-economic status.

Table S2 Diseases List

**Diseases**	**Psychiatric**	**Neurological**	**Cardiovascular**	**Total**	**Prevalence in Spain**(20)
Hypertension*				32·0%	31·1%
Heart problems			× 68·4%	3·5%	2·9%
Cholesterol*				57·3%	
Diabetes or blood sugar				2·0%	5·71%
Liver problems (hepatitis)				1·7%	
Cirrhosis				0·5%	0·8%
Anxiety	× 56·8%			11·2%	5·15%
Depression	× 43·2%			8·5%	7·18%
Loss of memory		× 74·6%		4·4%	-
Pancreatic problem				0·9%	0·13

Heart Attack		× 13·5%	0·7%	2·37%
Cerebral infarction		× 18·1%	0·9%	0·3
Sleep Apnea			3·6%	-
Dementia	× 1·1%		0·1%	1·1%
Parkinson´s Disease	× 4·5%		0·3%	0·8
Mild Cognitive Impairment	× 19·8%		1·2%	-
Arthritis			8·1%	0·6%
Amyotrophic lateral sclerosis			0·3%	
Multiple Sclerosis			0·2%	0·15%
Renal Disease (chronic nephritis)			0·3%	5·67%
Epilepsy			0·6%	0·4%
Schizophrenia psychosis			0·3%	0·32%
Meningitis encephalitis			0·3%	0·04%
Cancer			3·7%	2·75%

*These medical conditions were not considered in the total diseases analysis.

Table S3. Indices to determine the best-fitting model between 2-6 latent clusters.

No. Clusters	Calinski-Harabasz	Calinski-Harabasz - M	BIC	AIC	Ray-Turi	Davies-Bouldin
2	**398·6422**	398·6422	-372174·2	-371627·2	-0·04078	-1·768
3	321·815	455·1163	-364963·6	-364146·1	-0·038	-1·745

4	273·9780	474·5437	-360453·1	-359365·2	-0·03641	-1·736
5	245·1882	**490·3765**	**-355929·7**	**-354571·2**	**-0·0345**	**-1·704**

Note. The Calinski-Harabasz index is the ratio of the sum of between-clusters variance and within-clusters variance. The higher the score, the denser and more separated clusters, thus the better the model fit.

Among these criteria, some should be maximized (high value denoting good partition) while others should be minimized (low value denoting good partition). To avoid this confusion, package KmL3D computes all criteria to be maximized. Thus, for all indices, a higher score indicates a better-fitting model.

The AIC is the Akaike Information Criterion whilst the BIC is the Bayesian Information Criterion. When models with different numbers of latent classes are compared, the model with the lowest (highest in this case due to maximization) AIC and BIC is normally chosen as the best-fitting model. This is because a lower value of the information criterion suggests a better balance between model fit and parsimony (21).

Table S4 Proportion of missingness for indicators included in the imputation model.

**Variables**		**N (%) missingness**
age		0 (0)
sex		0 (0)
education		0 (0)
CRQ	Baseline	3013 (100%)
	Follow-up 1	126 (4·2%)
	Follow-up 2	154 (5·1%)
	Follow-up 3	516 (17·1%)
	Follow-up 4	995(33%)
IPAQ	Baseline	3013 (100%)

	Follow-up 1	126 (4·2%)
	Follow-up 2	154 (5·1%)
	Follow-up 3	516 (17·1%)
	Follow-up 4	995(33%)
Lubben	Baseline	12 (0·4%)
	Follow-up 1	149 (4·9%)
	Follow-up 2	155 (5·1%)
	Follow-up 3	516 (17·1%)
	Follow-up 4	995(33%)
Jenkins	Baseline	11 (0·4%)
	Follow-up 1	143 (4·7%)
	Follow-up 2	155 (5·1%)
	Follow-up 3	516 (17·1%)
	Follow-up 4	995(33%)
Medas	Baseline	5 (0·2%)
	Follow-up 1	128 (4·2%)
	Follow-up 2	154 (5·1%)
	Follow-up 3	516 (17·1%)
	Follow-up 4	995(33%)
RYFF	Baseline	0(0)
	Follow-up 1	156 (5·2%)

	Follow-up 2	156 (5·2%)
	Follow-up 3	516 (17·1%)
	Follow-up 4	995(33%)
Audit	Baseline	3013 (100%)
	Follow-up 1	126 (4·2%)
	Follow-up 2	141 (4·7%)
	Follow-up 3	516 (17·1%)
	Follow-up 4	995(33%)
Package by year	Baseline	0(0)
	Follow-up 1	125 (4·1%)
	Follow-up 2	141 (4·7%)
	Follow-up 3	516 (17·1%)
	Follow-up 4	995(33%)
BMI	Baseline	0(0)
	Follow-up 1	44 (1·5%)
	Follow-up 2	140 (4·6%)
	Follow-up 3	516 (17·1%)
	Follow-up 4	995 (33%)

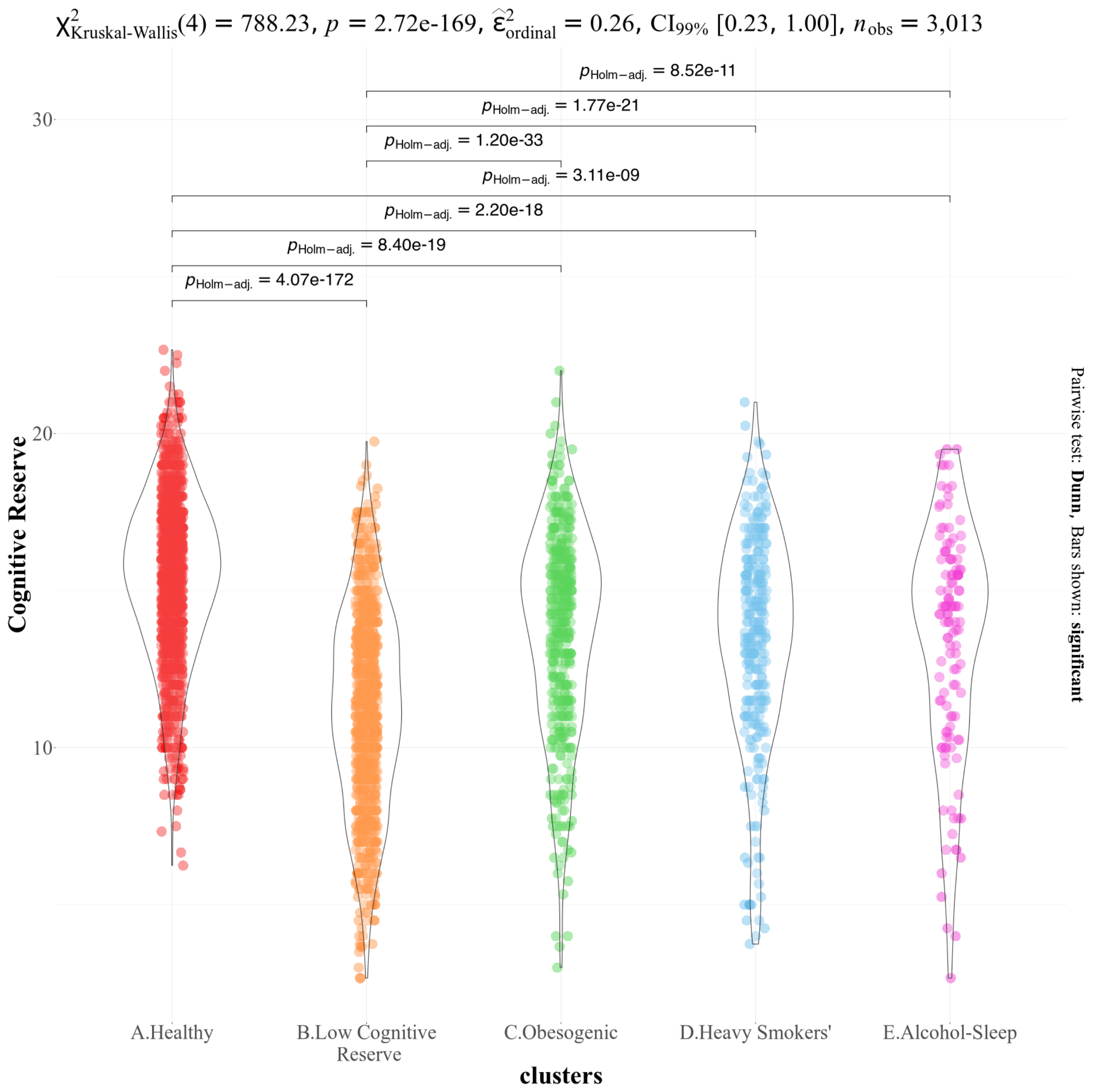

Supplementary Figure 1 . Comparison of Cognitive Reserve mean score across clusters.

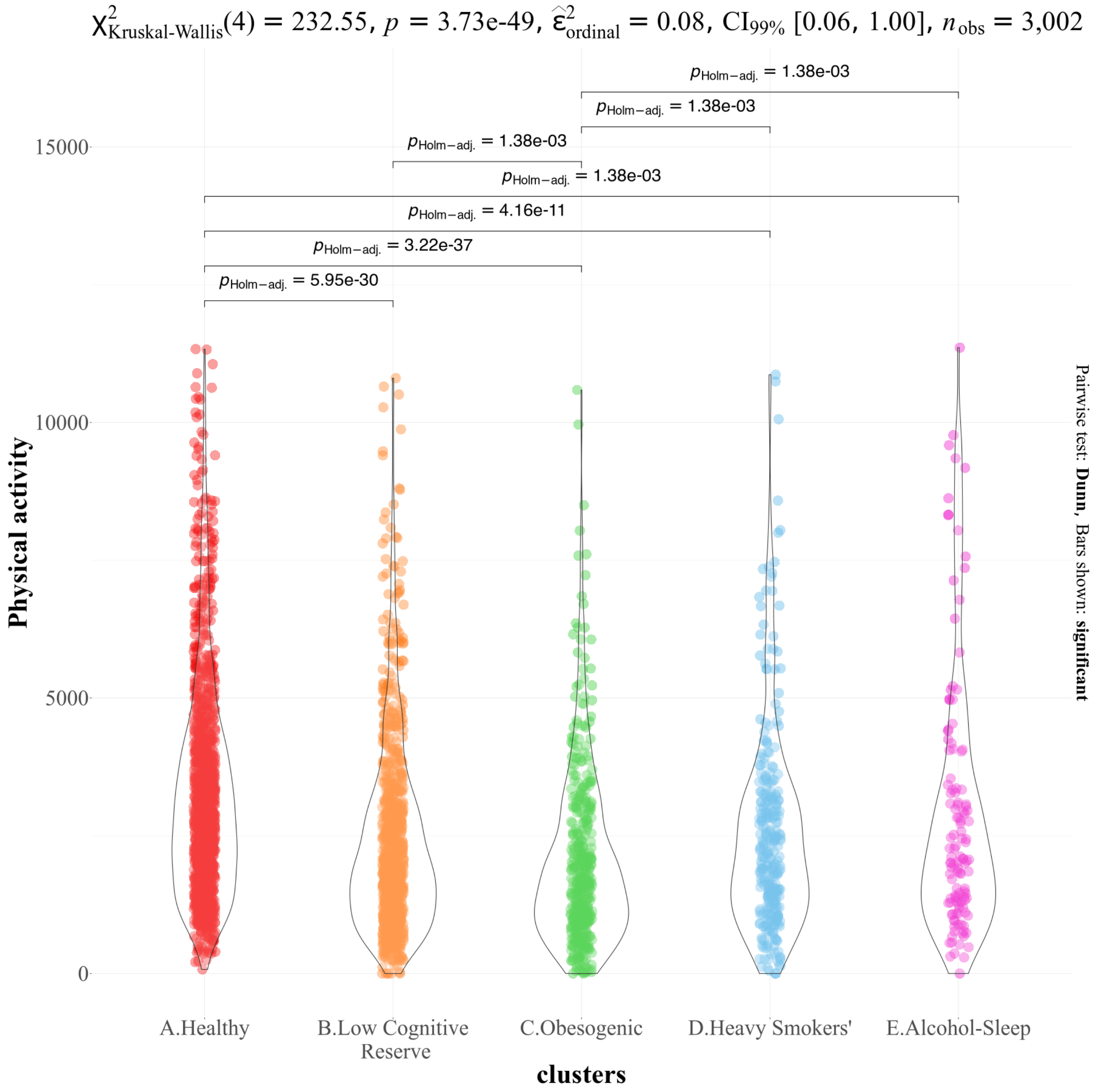

Supplementary Figure 2 . Comparison of physical activity mean score across clusters.

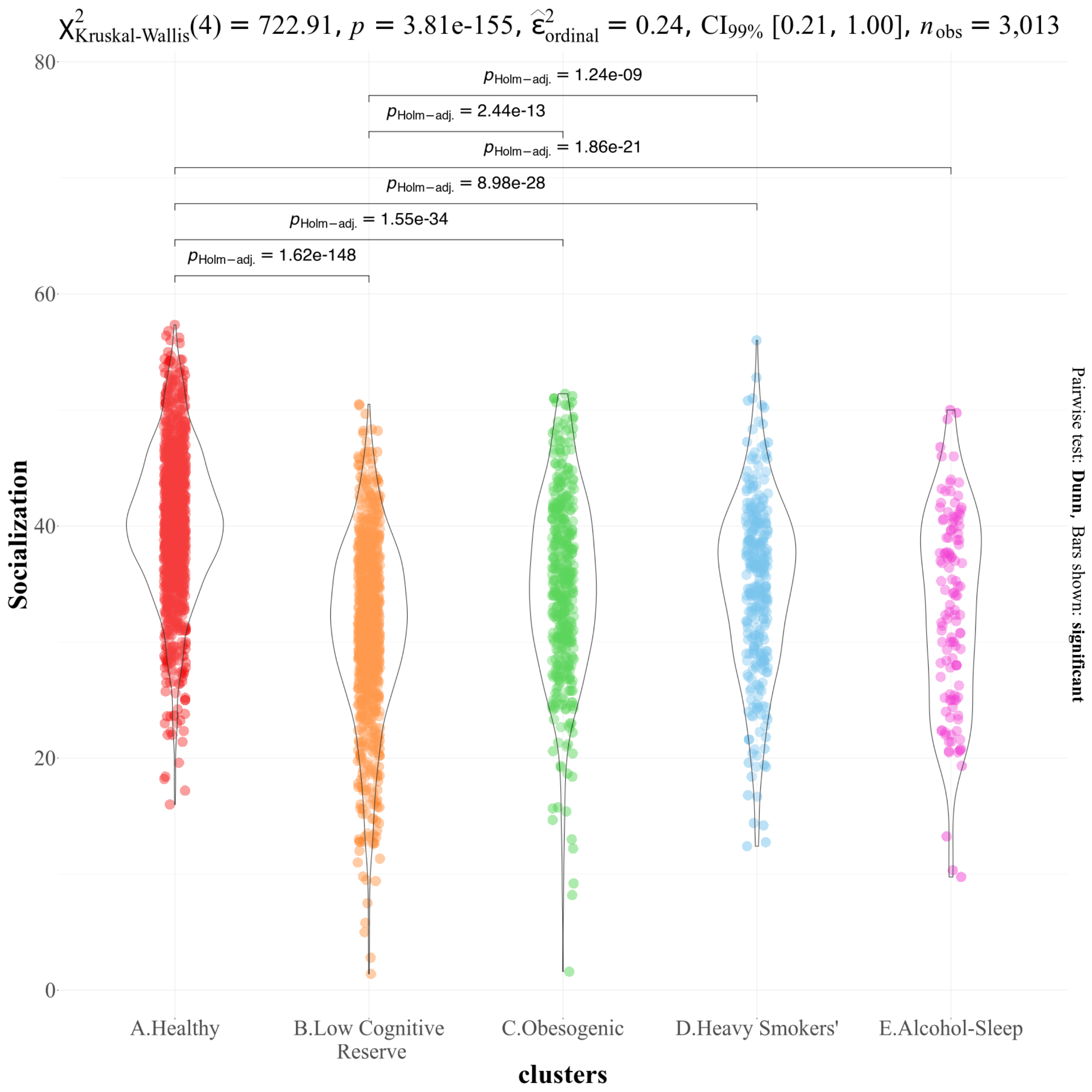

Supplementary Figure 3. Comparison of socialization mean score across clusters.

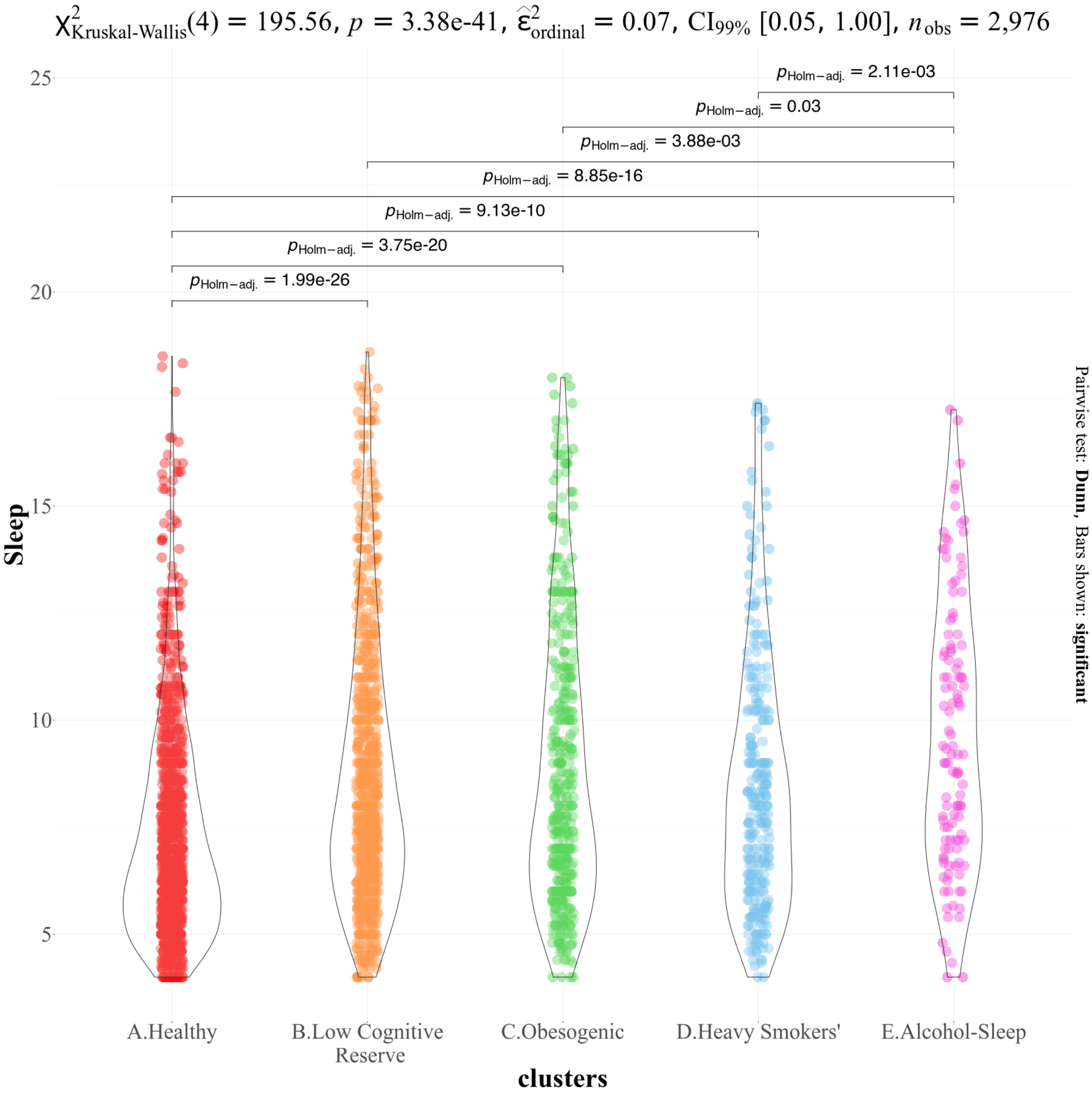

Supplementary Figure 4. Comparison of Sleep mean score across clusters.

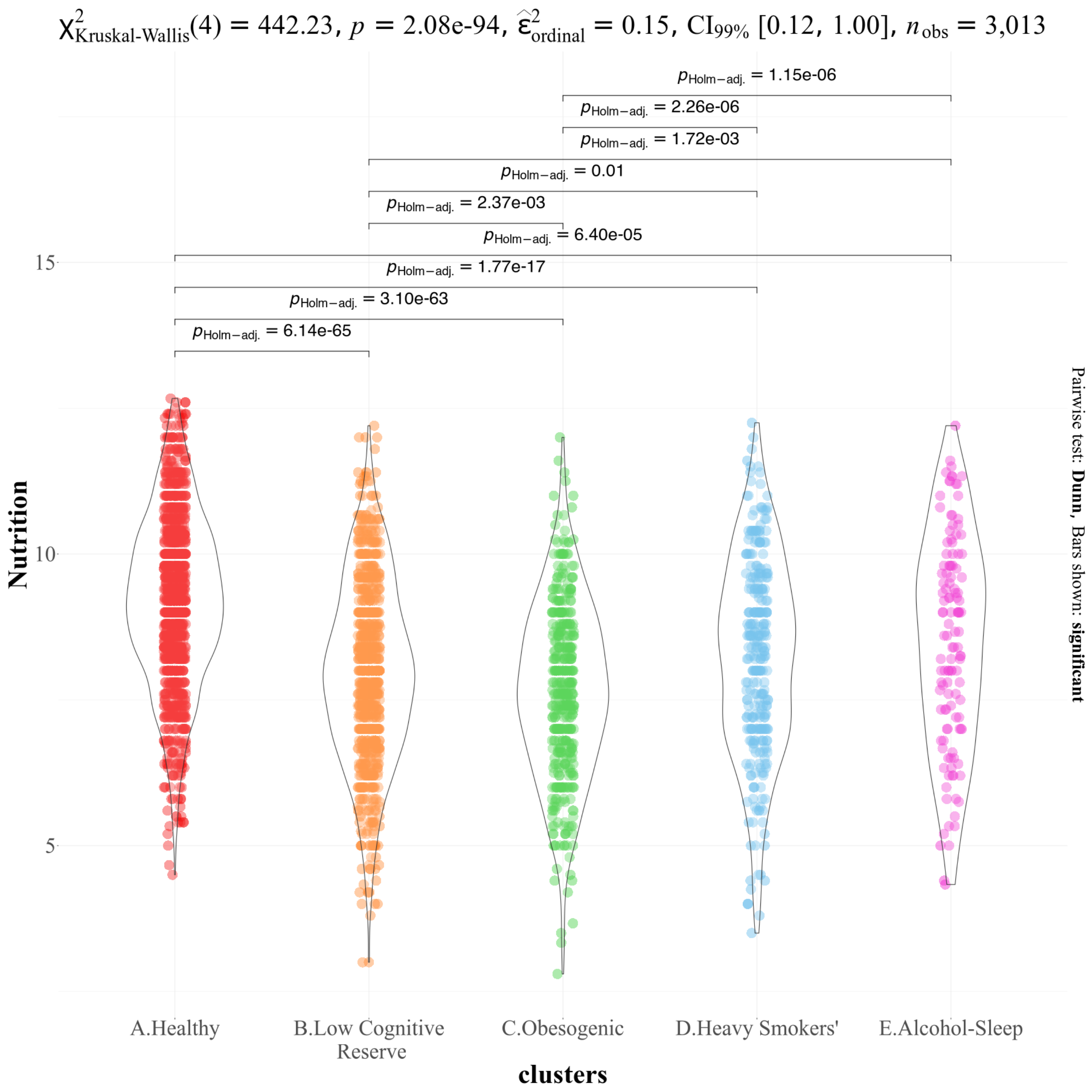
χ²Kruskal-Wallis(4) = 442.23, p = 2.08e-94, ε̂²ordinal = 0.15, CI99% [0.12, 1.00], nobs = 3,013
pHolm−adj. = 1.15e-06
pHolm−adj. = 2.26e-06
pHolm−adj. = 1.72e-03
pHolm−adj. = 0.01
pHolm−adj. = 2.37e-03
pHolm−adj. = 6.40e-05
pHolm−adj. = 1.77e-17
pHolm−adj. = 3.10e-63
pHolm−adj. = 6.14e-65
Nutrition
15
10
5
A.Healthy
B.Low Cognitive Reserve
C.Obesogenic
D.Heavy Smokers'
E.Alcohol-Sleep
clusters
Pairwise test: Dunn, Bars shown: significant

Supplementary Figure 5. Comparison of Nutrition mean score across clusters.

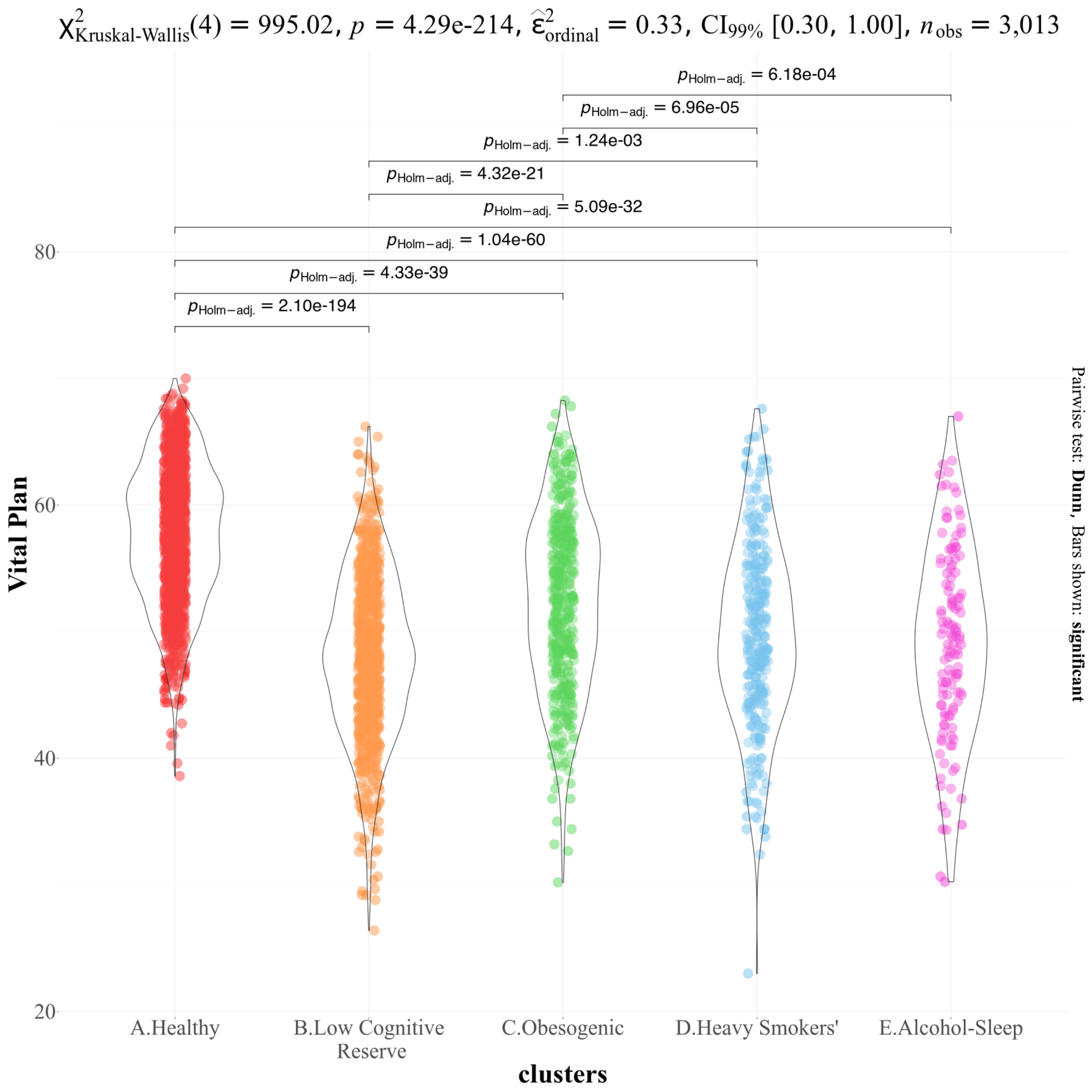

Supplementary Figure 6. Comparison of Vital Plan mean score across clusters.

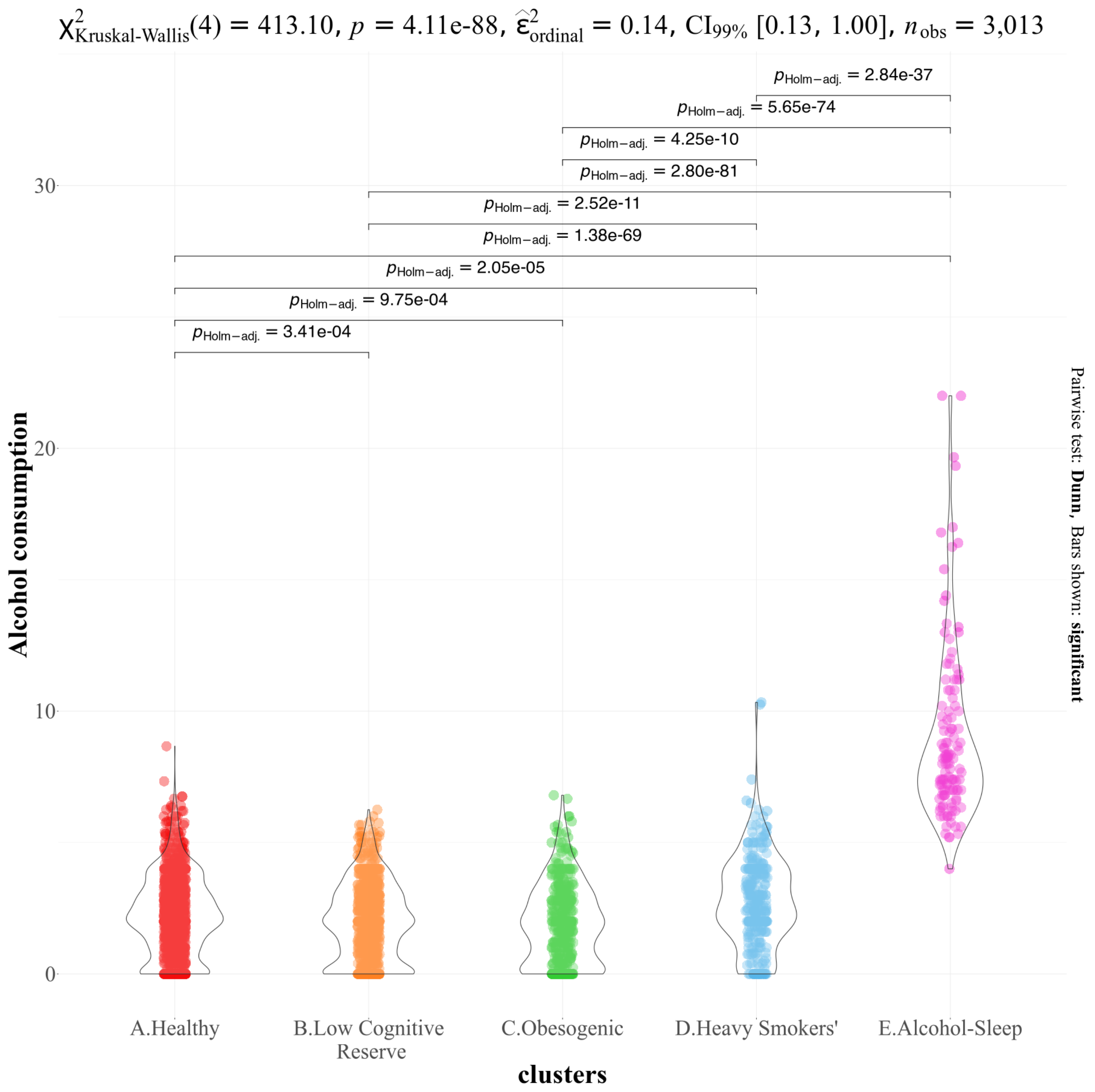

Supplementary Figure 7. Comparison of Alcohol consumption mean score across clusters.

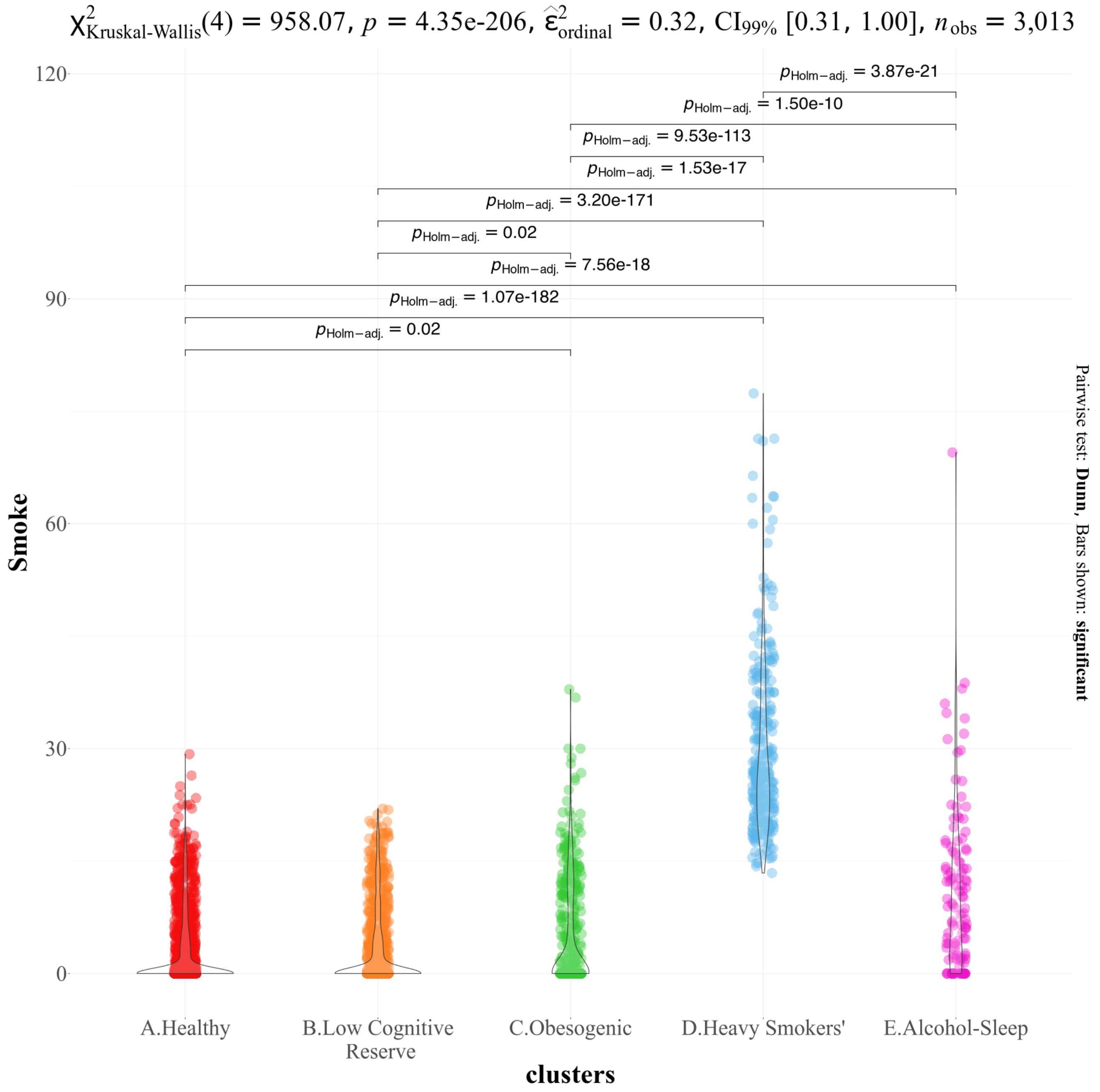

Supplementary Figure 8. Comparison of tobacco use mean score across clusters.

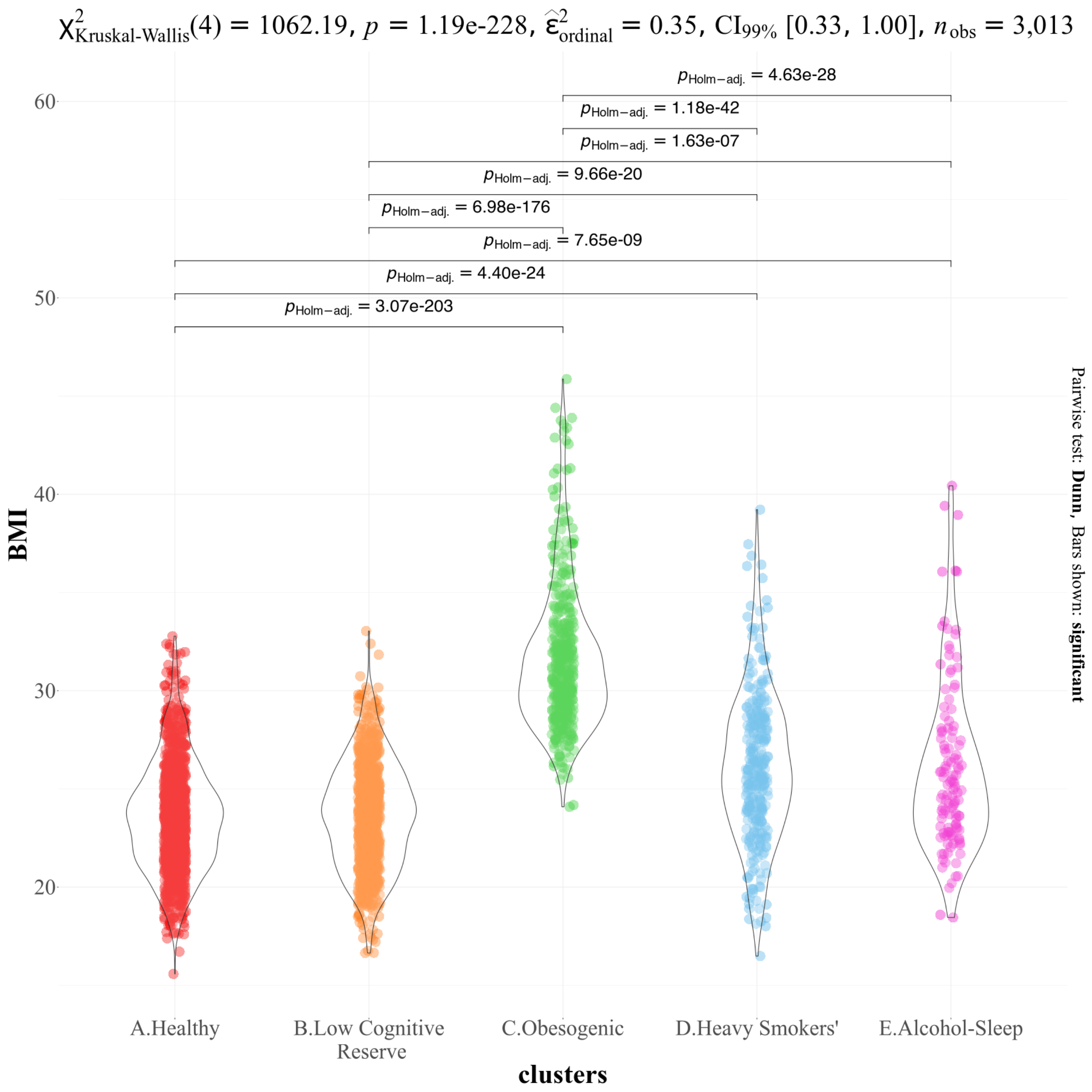

Supplementary Figure 9. Comparison of BMI mean score across clusters.

2 References

1. Jenkins CD, Stanton BA, Niemcryk SJ, Rose RM. A scale for the estimation of sleep problems in clinical research. J Clin Epidemiol. 1988;41(4):313-21.

2. Schröder H, Fitó M, Estruch R, Martínez-González MA, Corella D, Salas-Salvadó J, et al. A Short Screener Is Valid for Assessing Mediterranean Diet Adherence among Older Spanish Men and Women. J Nutr. 1 de junio de 2011;141(6):1140-5.

3. Ryff CD. Psychological Well-Being in Adult Life. Curr Dir Psychol Sci. 1 de agosto de 1995;4(4):99-104.

4. Lubben JE. Assessing social networks among elderly populations. Fam Community Health J Health Promot Maint. 1988;11:42-52.

5. Rami L, Valls-Pedret C, Bartrés-Faz D, Caprile C, Solé-Padullés C, Castellvi M, et al. Cognitive reserve questionnaire. Scores obtained in a healthy elderly population and in one with Alzheimer's disease. Rev Neurol. 16 de febrero de 2011;52(4):195-201.

6. Conigrave KM, Saunders JB, Reznik RB. Predictive capacity of the AUDIT questionnaire for alcohol-related harm. Addiction. noviembre de 1995;90(11):1479-85.

7. Craig CL, Marshall AL, Sjöström M, Bauman AE, Booth ML, Ainsworth BE, et al. International Physical Activity Questionnaire: 12-Country Reliability and Validity. Med Sci Sports Exerc. agosto de 2003;35(8):1381-95.

8. Ader D. Developing the Patient-Reported Outcomes Measurement Information System (PROMIS). Med Care. 1 de mayo de 2007;45:S1-2.

9. Kroenke K, Spitzer RL, Williams JBW, Löwe B. An ultra-brief screening scale for anxiety and depression: the PHQ-4. Psychosomatics. 2009;50(6):613-21.

10. Fieo R, Ocepek-Welikson K, Kleinman M, Eimicke JP, Crane PK, Cella D, et al. Measurement Equivalence of the Patient Reported Outcomes Measurement Information System® (PROMIS®) Applied Cognition - General Concerns, Short Forms in Ethnically Diverse Groups. Psychol Test Assess Model. 2016;58(2):255-307.

11. Brown TA, Chorpita BF, Korotitsch W, Barlow DH. Psychometric properties of the Depression Anxiety Stress Scales (DASS) in clinical samples. Behav Res Ther. 1 de enero de 1997;35(1):79-89.

12. Welcome to IPAQ [Internet]. [citado 23 de noviembre de 2022]. International Physical Activity Questionnaire. Disponible en: https://sites.google.com/site/theipaq/home

13. Chen S. Historical and Global Perspectives on Social Policy and "Aging in Community". Ageing Int. 1 de marzo de 2012;37(1):1-15.

14. Tibubos AN, Zenger M, Schmalbach B, Beutel ME, Brähler E. Measurement invariance, validation and normative data of the Jenkins Sleep Scale-4 (JSS-4) in the German general population across the life span. J Psychosom Res. marzo de 2020;130:109933.

15. Bittner N, Jockwitz C, Franke K, Gaser C, Moebus S, Bayen UJ, et al. When your brain looks older than expected: combined lifestyle risk and BrainAGE. Brain Struct Funct. abril de 2021;226(3):621-45.

16. Duriez Q, Crivello F, Mazoyer B. Sex-related and tissue-specific effects of tobacco smoking on brain atrophy: assessment in a large longitudinal cohort of healthy elderly. Front Aging Neurosci [Internet]. 3 de noviembre de 2014 [citado 24 de julio de 2023];6. Disponible en: http://journal.frontiersin.org/article/10.3389/fnagi.2014.00299/abstract

17. Franklin TR, Wetherill RR, Jagannathan K, Johnson B, Mumma J, Hager N, et al. The Effects of Chronic Cigarette Smoking on Gray Matter Volume: Influence of Sex. Zang YF, editor. PLoS ONE. 4 de agosto de 2014;9(8):e104102.

18. Karama S, Ducharme S, Corley J, Chouinard-Decorte F, Starr JM, Wardlaw JM, et al. Cigarette smoking and thinning of the brain's cortex. Mol Psychiatry. junio de 2015;20(6):778-85.

19. Goldberg LR. The development of markers for the Big-Five factor structure. Psychol Assess. 1992;4:26-42.

20. Global Health Data Exchange (GHDx) [Internet]. [citado 16 de mayo de 2023]. Institute of Health Metrics and Evaluation. Disponible en: https://vizhub.healthdata.org/gbd-results

21. Lanza ST, Rhoades BL. Latent Class Analysis: An Alternative Perspective on Subgroup Analysis in Prevention and Treatment. Prev Sci. 1 de abril de 2013;14(2):157-68.
